# Sex-differences in COPD: from biological mechanisms to therapeutic considerations

**DOI:** 10.3389/fmed.2024.1289259

**Published:** 2024-03-20

**Authors:** Kathryn M. Milne, Reid A. Mitchell, Olivia N. Ferguson, Alanna S. Hind, Jordan A. Guenette

**Affiliations:** ^1^Centre for Heart Lung Innovation, The University of British Columbia and Providence Research, St. Paul’s Hospital, Vancouver, BC, Canada; ^2^Division of Respiratory Medicine, The University of British Columbia, Vancouver, BC, Canada; ^3^Department of Physical Therapy, The University of British Columbia, Vancouver, BC, Canada

**Keywords:** chronic obstructive pulmonary disease, dyspnea, exercise physiology, sex-differences, exercise testing

## Abstract

Chronic obstructive pulmonary disease (COPD) is a heterogeneous respiratory condition characterized by symptoms of dyspnea, cough, and sputum production. We review sex-differences in disease mechanisms, structure-function-symptom relationships, responses to therapies, and clinical outcomes in COPD with a specific focus on dyspnea. Females with COPD experience greater dyspnea and higher morbidity compared to males. Imaging studies using chest computed tomography scans have demonstrated that females with COPD tend to have smaller airways than males as well as a lower burden of emphysema. Sex-differences in lung and airway structure lead to critical respiratory mechanical constraints during exercise at a lower absolute ventilation in females compared to males, which is largely explained by sex differences in maximum ventilatory capacity. Females experience similar benefit with respect to inhaled COPD therapies, pulmonary rehabilitation, and smoking cessation compared to males. Ongoing re-assessment of potential sex-differences in COPD may offer insights into the evolution of patterns of care and clinical outcomes in COPD patients over time.

## 1 Introduction

Chronic obstructive pulmonary disease (COPD) is a heterogeneous respiratory condition with hallmark chronic symptoms that include dyspnea, cough, and sputum production. It is characterized by airway remodeling and lung parenchymal destruction, resulting from a combination of environmental exposures and individual factors that ultimately alter the trajectory of normal lung development and aging, manifesting in disease (1). COPD is currently defined by the combination of symptoms, risk factors for disease, and airflow obstruction measured on spirometry (1). Dyspnea, reduced exercise capacity, and low quality of life frequently characterize the lives of COPD patients. The estimated global prevalence of COPD is approximately 12% and the prevalence and burden of COPD is increasing (1, 2). Due to multiple factors, the prevalence of COPD in females has increased and the number of females diagnosed with COPD in the United States now outnumbers males (3). COPD has also become the leading cause of death among female smokers (4). In females with COPD, dyspnea severity is greater and associated with increased morbidity compared to males (5–7). By comparison, data are mixed with some studies demonstrating that either males or females may be more likely to have symptoms of cough (8, 9). We review biological mechanisms underpinning sex-differences in COPD, the impacts of dyspnea in females with COPD, and structure-function-symptom interactions uncovered through use of imaging and cardiopulmonary exercise testing. Finally, we consider available evidence of clinical outcomes and treatment in females with COPD.

## 2 Sex in COPD

Sex is defined as the different biological and physiological characteristics of females, males, and intersex persons (10). Gender refers to the socially constructed characteristics of women and men that determine roles and relationships across an individual lifetime (10, 11). Understanding sex- and gender-differences and similarities in COPD is increasingly relevant given the growing focus on “treatable traits” and phenotyping patients (12, 13). There is mounting awareness that COPD is a heterogenous disease and that sex may play a role in the symptoms, clinical presentation, and outcomes of patients. The understanding of COPD in females and women has increased in recent decades, offering insights into observed differences in symptoms and clinical outcomes.

Biological differences between females and males include differences in airway growth leading to differential susceptibility to inhaled substances such as tobacco smoke, alterations in inflammatory responses in the lungs, and hormonal factors (14). Smaller airways relative to lung volume has been described in females (15). As a consequence, particle deposition from noxious substances, including cigarette smoke, may be greater in the proximal airways of females (16). Females experience greater small airways disease compared to males for a similar tobacco smoke exposure (5). Female smokers also experience a faster annual loss of forced expiratory volume in 1 s (FEV_1_) than male smokers (17). Female smokers tend to present at a younger age and lower pack-year smoking history, compared to males with the same degree of lung function impairment (18). This suggests that females may be more susceptible to the effects of cigarettes compares to males (19). Susceptibility to air contaminants is not limited to cigarettes and often non-smoking COPD patients are female (20). Exposures and risk factors associated with non-smoking COPD vary by geographic region and include exposure to biomass fuels, passive smoking, occupational exposures, infections, and air pollution (20–22).

The greater degree of small airways disease in females and increased emphysema in males may have a basis in sex hormones (11). In humans, testosterone level is associated with higher FEV_1_ after adjustment for age, height, and smoking (23). In mice models, females develop more small airways disease compared to males who develop more emphysema for the same level of cigarette smoke exposure (24). In female mice treated with tamoxifen or ovariectomy, greater emphysema developed, suggesting that estrogen may contribute to these observed sex-differences in mice (24). The Multi-Ethnic Study of Atherosclerosis Lung Study observed an association between male sex and paraseptal emphysema subtype visualized on computed tomography (CT) chest imaging (25) and an association between male sex and a diffuse emphysema subtype has also been described in a machine learning analysis of the Subpopulations and Intermediate Outcome Measures in COPD Study (26).

Despite the aforementioned insights, defining the role of sex-hormones in COPD and changes throughout the life cycle such as menopause in humans remains complex. Interestingly, early menopause has been associated with a lower risk of airflow obstruction, although other pulmonary function abnormalities such as a non-specific restrictive pattern, may develop in the peri-menopause period (27). Unlike the study of sex in respiratory health and disease, there is limited evidence to date regarding the potential role of gender in lung disease, as many studies to date have not included systematic gender assessment distinct from considering biological sex.

## 3 Dyspnea in females with COPD

Females with COPD experience more dyspnea compared to males, matched for relative lung function (14). Females less than 65 years of age have a higher exacerbation risk and higher odds of severe airflow obstruction in comparison to males (28). Females with COPD also experience increased depression and anxiety as well as reduced quality of life in comparison to their male counterparts (29–31). Importantly, dyspnea is strongly associated with depression in COPD (29). Anxiety and depression are associated with reduced smoking cessation (32), increased dyspnea (33), and reduced sleep quality (34, 35). Even after controlling for percent predicted lung function, age, smoking history, and extent of emphysema on chest CT, females still experience a greater dyspnea burden, more depression, and reduced quality of life relative to males (5). Female sex, depression, and anxiety are associated with increased risk of exacerbations and mortality (36–39). Recent population-based studies have demonstrated that sex-differences in dyspnea are accounted for by sex-differences in absolute values of lung function measurements [FEV_1_, forced vital capacity (FVC) or diffusing capacity of the lungs for carbon monoxide (D_*L*_CO)] (40–42). The complex interrelationships between dyspnea, mental health symptoms, and clinical outcomes are likely multidirectional. Dyspnea is central to the experience of both females and males with COPD, emphasizing the importance of understanding the underlying mechanisms of this symptom.

## 4 Structure-function-symptom relationships underpinning dyspnea in COPD

Quantitative chest CT scans enable sex-based comparisons of lung structure in people with COPD. Imaging studies using chest CT scans have demonstrated that females with COPD, matched for percentage predicted FEV_1_, tend to have smaller airways than males as well as a lower burden of parenchymal lung tissue destruction (5). Smaller airways on CT are measured as reduced airway luminal area and diameter, reduced wall thickness, and increased airway wall area (43, 44). Female smokers without established COPD as well as females with severe COPD have less emphysema compared to males (5, 45). Although the precise mechanisms underlying anatomical differences are an area of ongoing research, examining the functional consequences of these structural differences, relative to health, under the stress of exercise with cardiopulmonary exercise testing (CPET), enables integration of structural differences with differences in symptoms, specifically exertional dyspnea.

The current understanding of mechanisms of dyspnea in females with COPD is partially informed by data examining sex-differences in dyspnea in healthy individuals. The Burden of Obstructive Lung Diseases international population-based study describes a greater prevalence of dyspnea in females compared to males, including in a subpopulation of participants without self-reported diseases associated with dyspnea or abnormal spirometry (46). Males have larger conducting airways and lung volumes in comparison to females, even when accounting for differences in height (47–49). Healthy females also have reduced respiratory muscle strength compared to height-matched males (50). These differences may predispose females to developing greater ventilatory abnormalities and increases in both neural respiratory drive, the signal to breathe from the brain to the respiratory muscles, and dyspnea intensity during exercise relative to males (51, 52). There are also sex differences in qualitative descriptors of dyspnea, with healthy females being more likely to select unpleasant dyspnea descriptors related to inspiratory difficulty, shallow breathing, and unsatisfied inspiration than males (52). Sex-differences in dyspnea quality in health are likely a manifestation of relatively greater respiratory mechanical constraints and neural respiratory drive at a given absolute ventilation in females (51, 52).

Dyspnea in COPD is closely related to the physiological consequences of the disease and can be systematically studied using CPET. Airway remodeling and lung parenchymal destruction in COPD leads to airflow limitation, increased lung compliance, and impaired gas exchange. During CPET, the consequences of these abnormalities manifest as dynamic hyperinflation, respiratory muscle weakness, ventilatory inefficiency, and hypoxemia (53–55). Abnormal responses to exercise provide a powerful stimulus for increased neural respiratory drive to the diaphragm in an effort to increase ventilation commensurate with the metabolic demands of exercise (53–55). Neural respiratory drive can be estimated using diaphragm electromyography during CPET and is highly correlated with dyspnea in COPD (53–55).

**FIGURE 1:**
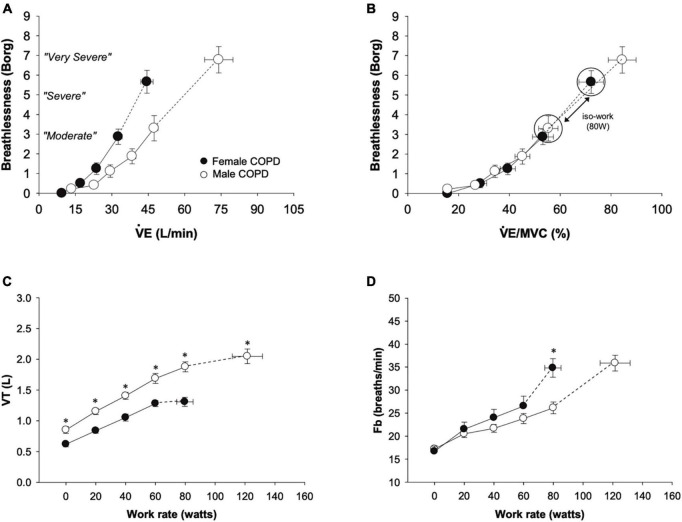
Comparison from rest to peak exercise between males and females with mild COPD in **(A)** dyspnea, **(B)** dyspnea relative to ventilation as a percentage of estimated maximum ventilatory capacity, **(C)** tidal volume, and **(D)** breathing frequency. Females with COPD experienced greater dyspnea for a given absolute ventilation during exercise and had reduced tidal volume expansion and consequent increased breathing frequency compared to males. At a work rate of 80 W (the highest absolute work rate comparison between males and females with COPD), the V_*E*_/MVC ratio and dyspnea were greater in females. Values are mean ± SE. *p < 0.05. Fb, breathing frequency; MVC, maximum ventilatory capacity; V_*E*_, minute ventilation; V_*T*_, tidal volume. Reprinted from Guenette et al. (6) with permission from Elsevier.

In a detailed physiology study in females and males with mild COPD, matched for % predicted FEV_1_, females experienced significantly greater dyspnea intensity ratings during exercise (Figure 1A), constraints on tidal volume expansion (Figure 1C), and a more rapid breathing pattern (Figure 1D) at a given absolute work rate in comparison to males (6). However, when dyspnea was assessed relative to maximum ventilatory capacity (Figure 1B), sex differences in dyspnea between males and females with COPD disappeared, suggesting that increased intensity of exertional dyspnea in females is largely driven by females using a greater fraction of their ventilatory capacity to achieve the same absolute exercise intensity and ventilation compared with males (6).

Differences in absolute lung function between males and females in relation to dyspnea has also been examined in several recent population-based studies. The European Community Respiratory Health Survey, a general population-based study, found that dyspnea was twice as common in females and was explained by differences in absolute FEV_1_ and FVC values, demonstrating that lower absolute lung function accounts for dyspnea in females (41). In the population-based Swedish CArdioPulmonarybioImage Study, similar results were found with respect to absolute diffusing capacity and static lung volumes as well as in obese females (42, 56). Similar findings have been described in individuals with COPD in the COPDGene study, demonstrating that sex-based differences in absolute FEV_1_ accounted for the difference in dyspnea between females and males (40).

Taken together, detailed physiological studies and larger scale population-based studies demonstrate that differences in exertional dyspnea intensity between females and males are largely explained by differences in absolute measures of lung function and the relatively higher ventilation needed in females to perform a given absolute task compared to males. However, the underlying physiologic explanations for differences in dyspnea quality and unpleasantness across the spectrum of COPD disease severity and any potential association with observed higher anxiety and depression in females with COPD, remains unknown and an important area for future research.

## 5 COPD therapies

Major classes of inhaled medications for COPD include long-acting muscarinic antagonists (LAMA), long-acting beta agonists (LABA), combination LABA-LAMA, and combination inhaled corticosteroid (ICS)-LABA. A recent systematic review of randomized controlled trials and observational studies found that there is limited data directly comparing treatment effectiveness between sexes in COPD; however, there is evidence from retrospective and sub-group analyses of large randomized controlled trials (57). When clinical trials report sex-differences, they are frequently not found; however, some trials may not be powered to detect differences in treatment response between females and males. Tiotropium demonstrates a similar improvement in exercise capacity, trough FEV_1_, reduced exacerbations, and improved quality of life in males and females (58, 59). Females experience improvements in dyspnea with LABA-LAMA similar to males in comparison to either LAMA or LABA alone (60, 61). Comparing LABA-LAMA to ICS-LABA, improvement in FEV_1_ are observed in both males and females to varying degrees (60–62). Only males had a significant reduction in exacerbations (62), but females had better responses with regards to dyspnea and quality of life with LABA-LAMA compared to ICS-LABA (61). Taken together, there is no evidence to support treating females with COPD differently from their male peers in terms of inhaled therapies.

There is similarly no evidence to support differential non-pharmacologic management of individuals with COPD based on sex. Despite greater benefits of smoking cessation with respect to lung function in females compared to males, females find it harder to quit and are more likely to relapse (63). However, upon smoking cessation, the gain in FEV_1_ in female ex-smokers is greater than males (63). Long term oxygen therapy in females with COPD affords benefits with respect to survival similar to males (64, 65). There are no established sex-differences in exercise capacity and quality of life improvement following pulmonary rehabilitation in females compared to males, although benefits of pulmonary rehabilitation may not be sustained in females (66).

## 6 COPD outcomes in females

Previously described significant disparities in the accurate diagnosis and clinical outcomes of females with COPD may be changing (67–69). Ongoing prospective re-evaluation of potential sex-differences in females and women with COPD is valuable to understanding how patterns of care may change over time. Females with COPD also present with different comorbidities compared to their male counterparts. Females more often have heart failure, osteoporosis, and diabetes (70). Interestingly, different combinations of comorbidities in COPD are associated with mortality between sexes (71). Healthcare related costs for those living with COPD account for the majority of expenditures in respiratory disease in Europe and are expected to increase in the United States (1). Females incur greater healthcare costs than males and lose more quality-adjusted life years (72). Females with COPD have a higher BODE index (body mass index, airflow obstruction, dyspnea, and exercise capacity) suggesting a worse prognosis (73). Females have also been at greater risk of hospitalization and death (74). Female sex has additionally been identified as a risk factor for acute exacerbations of COPD in the large COPDGene cohort study (39).

## 7 Conclusion

Sex and gender have the potential to influence biological mechanisms, symptoms, and clinical outcomes. Several mechanisms may contribute to sex-differences in COPD including differences in airway and lung structure, differential consequent susceptibility to inhaled pollutants, and sex-hormones. Females with COPD experience significantly greater dyspnea compared to males. In mild COPD, females have greater respiratory mechanical constraints at a lower work rate compared to males, which is associated with greater neural respiratory drive and dyspnea (6). However, differences in exertional dyspnea intensity have been demonstrated to be related to differences in absolute measures of lung function and consequent differences in ventilatory capacity between sexes.

Insights into sex-differences and similarities in lung structure, exercise physiology, and responses to mainstay COPD therapies has advanced over the past decade. In contrast, our understanding of gender-differences in COPD remains limited, as studies either frequently neglect to systematically assess gender, or conflate gender related factors with sex. Considering the multidimensional nature of dyspnea, our collective understanding of differences in dyspnea quality between sexes and genders in COPD remains an area of future research.

## Author contributions

KM: Conceptualization, Writing – original draft, Writing – review & editing. RM: Writing – review & editing. OF: Writing – review & editing. AH: Writing – review & editing. JG: Conceptualization, Writing – original draft, Writing – review & editing.
